# Diabetes mellitus and latent tuberculosis infection: an updated meta-analysis and systematic review

**DOI:** 10.1186/s12879-023-08775-y

**Published:** 2023-11-08

**Authors:** Guozhong Zhou, Xin Guo, Shunli Cai, Yu Zhang, Yuanyuan Zhou, Rong Long, Yingchen Zhou, Hanse Li, Nan Chen, Chao Song

**Affiliations:** 1 Department of Science and Research, The Affiliated Anning First People’s Hospital of Kunming University of Science and Technology, Kunming, 650302 Yunnan Province China; 2 School of Basic Medical Sciences, Kunming Medical University, Kunming, 650051 Yunnan Province China; 3 Department of Endocrinology, The Affiliated Anning First People’s Hospital of Kunming University of Science and Technology, Kunming, 650302 Yunnan Province China; 4 Department of Endocrinology and Metabolism, Sixth Affliated Hospital of Kunming Medical University, The People’s Hospital of Yuxi City, Yunnan Province, Yuxi, 653100 China; 5 The School of Medicine, Kunming University, Kunming, 650214 China; 6 Department of Medical Imaging, The Affiliated Anning First People’s Hospital of Kunming University of Science and Technology, Kunming, 650302 Yunnan Province China

**Keywords:** Latent tuberculosis infection, Diabetes mellitus, Meta-analysis

## Abstract

**Background:**

Previous studies have demonstrated an association between diabetes mellitus (DM) and latent tuberculosis infection (LTBI). This study was conducted to update the current understanding of the association between DM and LTBI. By conducting a systematic review and meta-analysis using adjusted odds ratios (aOR) or risk ratios (aRR), we aimed to further explore the association between DM and LTBI and provide essential reference for future research.

**Methods:**

We conducted comprehensive searches in Embase, Cochrane Library, and PubMed without imposing any start date or language restrictions, up to July 19, 2022. Our study selection encompassed observational research that compared from LTBI positive rates in both DM and non-DM groups and reported aRR or aOR results. The quality of the included studies was assessed utilizing the Newcastle–Ottawa Scale. Pooled effect estimates were calculated using random-effects models, along with their associated 95% confidence intervals (CI).

**Results:**

We included 22 studies involving 68,256 subjects. Three cohort studies were eligible, with a pooled aRR of 1.26 (95% CI: 0.71–2.23). Nineteen cross-sectional studies were eligible, with a pooled aOR of 1.21 (95% CI: 1.14–1.29). The crude RR (cRR) pooled estimate for three cohort studies was 1.62 (95% CI: 1.03–2.57). Among the cross-sectional studies we included, sixteen studies provided crude ORs, and the crude OR (cOR) pooled estimate was 1.64 (95% CI: 1.36–1.97). In the diagnosis of diabetes, the pooled aOR of the HbA1c group was higher than that of self-reported group (pooled aOR: 1.56, 95% CI: 1.24–1.96 *vs.* 1.17, 95% CI: 1.06–1.28).

**Conclusion:**

Our systematic review and meta-analysis suggest a positive association between DM and LTBI. Individuals with DM may have a higher risk of LTBI compared to those without DM. These findings provide important insights for future research and public health interventions in managing LTBI in diabetic populations.

**Supplementary Information:**

The online version contains supplementary material available at 10.1186/s12879-023-08775-y.

## Introduction

Latent tuberculosis infection (LTBI) is a non-infectious, asymptomatic, persistent immune state following infection with *Mycobacterium tuberculosis* (TB) [1]. Patients with LTBI are at risk of reactivation if they become immunocompromised, causing progression to active symptomatic and highly contagious TB infection; therefore, LTBI is an important public health issue [2]. A quarter of the global population is estimated to have LTBI which comprises a large pool of potential TB patients [3].

Diabetes mellitus (DM) is a noncommunicable disease that occurs when the pancreas is unable to produce enough insulin hormones or when the body is unable to use insulin effectively [4]. Statistically, one in every 28 cases of DM dies, with an estimated 2 million people dying of DM each year [5]. Due to unhealthy lifestyles and rising trends in obesity, DM is expected to affect 578 million people worldwide by 2030 and 700 million by 2045 [6]. A previous systematic review and meta-analysis of data of 2.3 million people with TB globally, estimated that the prevalence of DM in patients with TB is around 15%, about twice that of the general population [7]. It has been demonstrated that those with DM are at increased risk of active TB [8]. DM has also been shown to be associated with LTBI in the meta-analysis.

In 2017, a meta-analysis by Lee and colleagues revealed that DM was associated with a small but statistically significant risk for LTBI, with an adjusted odds ratio (OR) of 1.18 (95% confidence interval [CI]: 1.06–1.30) [9]. In 2022, a meta-analysis by Liu and colleagues showed that the risk of LTBI in patients with DM was 60% greater than what was previously reported, with a crude OR of 1.55 (95% CI 1.30–1.84) [10]. While the association between DM and LTBI has been confirmed, the updated systematic review and meta-analysis conducted by Liu et al. relied on crude effect estimates for this association, which are susceptible to confounding factors. To overcome this limitation, we conducted an updated meta-analysis that exclusively included studies reporting adjusted effect estimates for the association between DM and LTBI.

## Methods

The study is registered in the PROSPERO database (CRD42022306589) and reported in accordance with Meta-analysis of Observational Studies in Epidemiology (MOOSE) guidelines [11]. We searched Embase, Cochrane Library, and PubMed through July 31, 2022, without a start date or language restrictions. Search terms included subject and keywords of DM and LTBI (Supplementary Table S1). To identify additional articles, we also searched bibliographic references of related works. We included observational studies that employed the tuberculin skin test (TST) or interferon-γ release assay (IGRA) for diagnosing LTBI, and utilized methods such as glycated hemoglobin and self-report for diagnosing DM.

### Selection criteria

The following inclusion criteria were applied: (1) observational studies (cohort, case–control, and cross-sectional); (2) used of either a TST and/or an IGRA for the diagnosis of LTBI; (3) compared from LTBI positive rates in both DM and non-DM groups and reported aRR or aOR results.

The following exclusion criteria were applied: (1) the population including patients with active TB; (2) observational studies providing only crude effect estimates of the association between DM and LTBI; (3) DM assessed as an adjusted but not an exposure factor; (4) abstracts, letters, case reports, or reviews.

Two investigators (GZZ and XG) independently screened article titles and abstracts retrieved from the literature search. Full texts of those potentially eligible studies were further assessed for final inclusion. A third investigator (NC) cross-checked extracted data, with disagreements resolved through a consensus.

### Study selection and data extraction

Data extraction was performed using a form that included the following fixed set of fields: title, author, year of publication, country or area, study type, patient demographics, TB burden, diagnostic method of LTBI, DM definition, and crude and adjusted effect sizes and their 95% CIs. Two investigators (GZZ and XG) independently extracted data from individual studies. A third investigator (NC) cross-checked extracted data, and disagreements were resolved through a consensus.

### Quality assessment

A modified version of a risk-of-bias tool used in a previous systematic review and the modified Newcastle–Ottawa scale for observational studies were used to assess the quality of included studies [12]. Cohort studies were scored using an 8-point scale to determine overall quality. Studies were classified based on their risk of drift, as follows: low (6–8), moderate (4–5), and high (< 4). Cross–sectional studies were classified based on risk of bias using a 7-point scale, as follows: low (5–7), medium (3–4), and high (< 3) (Supplementary material 2). Two investigators (GZZ and XG) independently assessed the methodological quality of the studies, with a third investigator (NC) independently reviewing their assessments. Disagreements were resolved by reaching a consensus.

### Data analysis

A random-effects model was used to calculate pooled results with a 95% CI. We performed Cochran's Q test using R to assess the heterogeneity of the included studies. The Egger’s test and the funnel plot were used to assess the publication bias [13–15]. We also conducted meta-regression and subgroup analysis to explore the heterogeneity of the articles. Potential factors encompass various aspects, including diverse methods for diagnosing diabetes, distinct approaches for diagnosing LTBI, variations in the study population, background prevalence of LTBI within the study population, bias of quality assessment and the TB burden, among others.

This meta-analysis was conducted using the “meta” package of R statistical software version 3.4.3. In addition, to facilitate data collation and analysis, cohort studies showing an association between DM and LTBI using an OR value, had the OR values converted to RR using a calculation [16].

## Results

### Study selection and characteristics

**Fig. 1:**
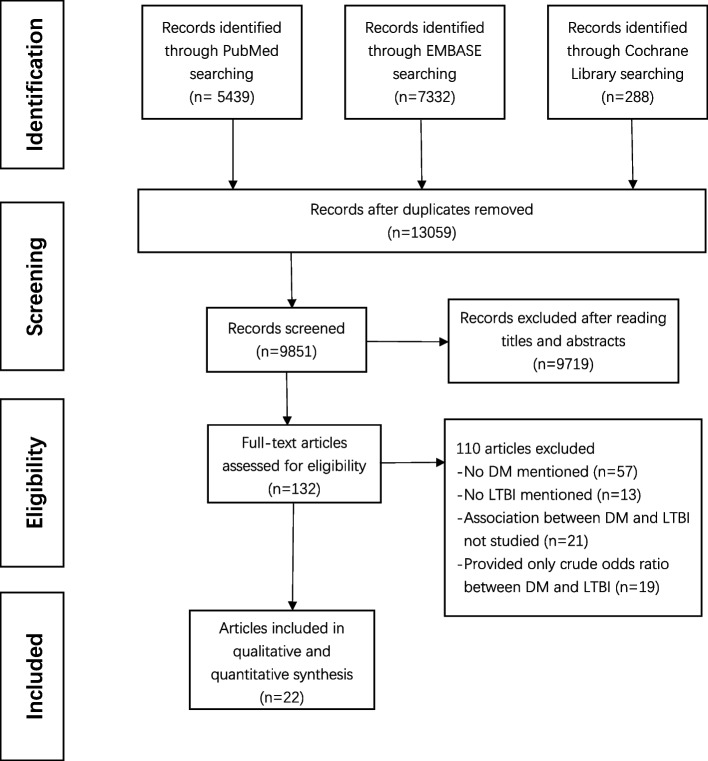
Flow chart of literature search. Abbreviations: DM, diabetes mellitus; LTBI, latent tuberculosis infection

We searched the Embase, Cochrane Library and PubMed databases for 13,059 records, excluding 3208 duplicates. We excluded 9,719 reviews, conference papers, animal experiments, case reports, and studies not related to the topic based on titles and abstracts. After excluding 110 articles that did not meet the inclusion and exclusion criteria, we ended up including 22 studies (Fig. 1). The final meta-analysis included 19 cross-sectional studies and 3 cohort studies. Basic characteristics of included studies are summarised in Table 1. The 22 studies included 68,256 individuals from nine countries. Among them, 19 had low and 3 had moderate risk of bias (Supplementary Figures S1 and 2).

**Table 1:** Characteristics of 22 observational studies in the review

Author and year	Study Type	Country	Study population (n)	Exclusion of active TB	LTBI prevalence (%)	DM prevalence (%)	Age (mean/ median)	LTBI diagnosis	DM diagnosis	aOR (95% CI)
Arnedo-Pena 2015 [17]	Cohort	Spain	Contacts (198)	Yes	9.1	2.0	37.5 ± 12.9	IGRA and TST	Unclear	aRR 4.40 (0.50–38.55)
Khawcharoenporn 2015 [18]	Cohort	Taiwan, China	HIV-positive (150)	Yes	24.0	4.0	40 (17–65)	IGRA	Interview and medical records	aRR 1.52 (0.38–3.24)
Wang 2012 [19]	Cohort	Taiwan, China	Household contacts (583)	Yes	30.2	2.9	Mean: 44.7	IGRA	Self-report	aRR 1.01 (0.44–1.84)
Alvarez 2014 [20]	Cross-sectional	Canada	Household contacts (185)	Yes	17.3	2.7	25 (1–85)	TST	Unclear	0.23 (0.02–2.65)
Arnedo-Pena 2015 [17]	Cross-sectional	Spain	Contacts (386)	Yes	23.3	3.6	35.4 ± 9.8	IGRA and TST	Unclear	1.71 (0.48–6.08)
Barron 2018 [21]	Cross-sectional	USA	Community residents (4958)	Yes	4.4	11.4	Unkonwn, age ≥ 20	IGRA	HbA1c	1.90 (1.15–3.14)
Bennet 2013 [22]	Cross-sectional	USA	Immigrants (4187)	Yes	19.7	4.2	Mean: 31.0	TST or IGRA	Self-report	1.58 (1.13–2.20)
Chan-Yeung 2006 [23]	Cross-sectional	China	Old age home residents (3605)	No	46.3	22.4	82.4 ± 8.3	TST	Medical records	1.15 (0.97–1.37)
Hensel 2015 [24]	Cross-sectional	USA	Refugees (702)	Yes	31.5	7.7	Mean: 36.1 (26.6–42.3)	IGRA	HbA1c	2.27 (1.15–4.48)
Jackson 2013 [25]	Cross-sectional	UK	Contacts (4730)	Yes	29.3	6.0	Unknown, age ≥ 16	IGRA	Self-report	1.15 (0.88–1.50)
Jackson 2019 [26]	Cross-sectional	UK	Community residents (9157)	Yes	27.7	8.3	NS	IGRA	Self-report	1.15 (1.02–1.30)
Koesoemadinata 2017 [27]	Cross-sectional	Indonesia	Household contacts (651)	No	NS	50.0	NS	IGRA	Interview and medical records	1.71 (1.19–2.45)
Kubiak 2019 [28]	Cross-sectional	India	Household contacts (1113)	Yes	54.4	6.2	36.8 ± 14.4	TST	Self-report	1.20 (0.99–1.45)
Lee 2010 [29]	Cross-sectional	Taiwan, China	Hemodialysis patients (83)	Yes	38.6	26.5	58.3 ± 14.9	IGRA	Self-report	0.58 (0.15–2.21)
Lin 2019 [30]	Cross-sectional	Taiwan, China	Community residents (3401)	Yes	19.6	86.7	61.5 ± 9.3	IGRA and TST	HbA1c	1.67 (1.18–2.38)
Liu 2020 [31]	Cross-sectional	China	Community residents (5405)	Yes	37.8	5.6	50 (40–61)	IGRA	Fasting blood glucose test	1.16 (0.88–1.51)
Martinez 2017 [32]	Cross-sectional	USA	Community residents (4215)	Yes	3.34	18.4	NS	IGRA	Self-report	1.50 (1.00–2.20)
Salindri 2021 [33]	Cross-sectional	USA	Community residents (132)	Yes	10.6	74.2	54 (49–60)	IGRA	HbA1c	0.45 (0.13–1.71)
Shu 2012 [34]	Cross-sectional	Taiwan, China	End-stage renal disease (407)	Yes	22.4	25.1	61.1 ± 13.1	IGRA	Self-report	0.89 (0.51–1.56)
Suwanpimolkul 2014 [35]	Cross-sectional	USA	Immigrants (22,227)	Yes	80.3	6.1	NS	TST or IGRA	Medical records	1.13 (0.97–1.33)
Swarna Nantha 2017 [36]	Cross-sectional	Malaysia	Non-communicable disease patients (763)	Yes	28.8	52.9	62.3 ± 10.0	TST	HbA1c	1.23 (0.81–1.86)
Ting 2014 [37]	Cross-sectional	Taiwan, China	High-risk (1018)^a^	Yes	29.1	14.4	Mean: 59.0	IGRA	Interview and medical records	1.11 (0.75–1.63)

*Abbreviations*: *DM* diabetes mellitus, *IGRA* interferon-γ release assay, *LTBI* latent tuberculosis infection, *TST* tuberculin skin test, *aOR* adjusted odds ratios, *aRR* adjusted risk ratios, *NS* Not specified

^a^High-risk: people with active TB contact, health care workers, and patients with malignancy, end-stage renal disease, liver cirrhosis, post-organ transplantation, autoimmune diseases, and fibro-calcified lesions suggestive of prior TB on chest radiogram

### Main outcome analysis

**Fig. 2:**
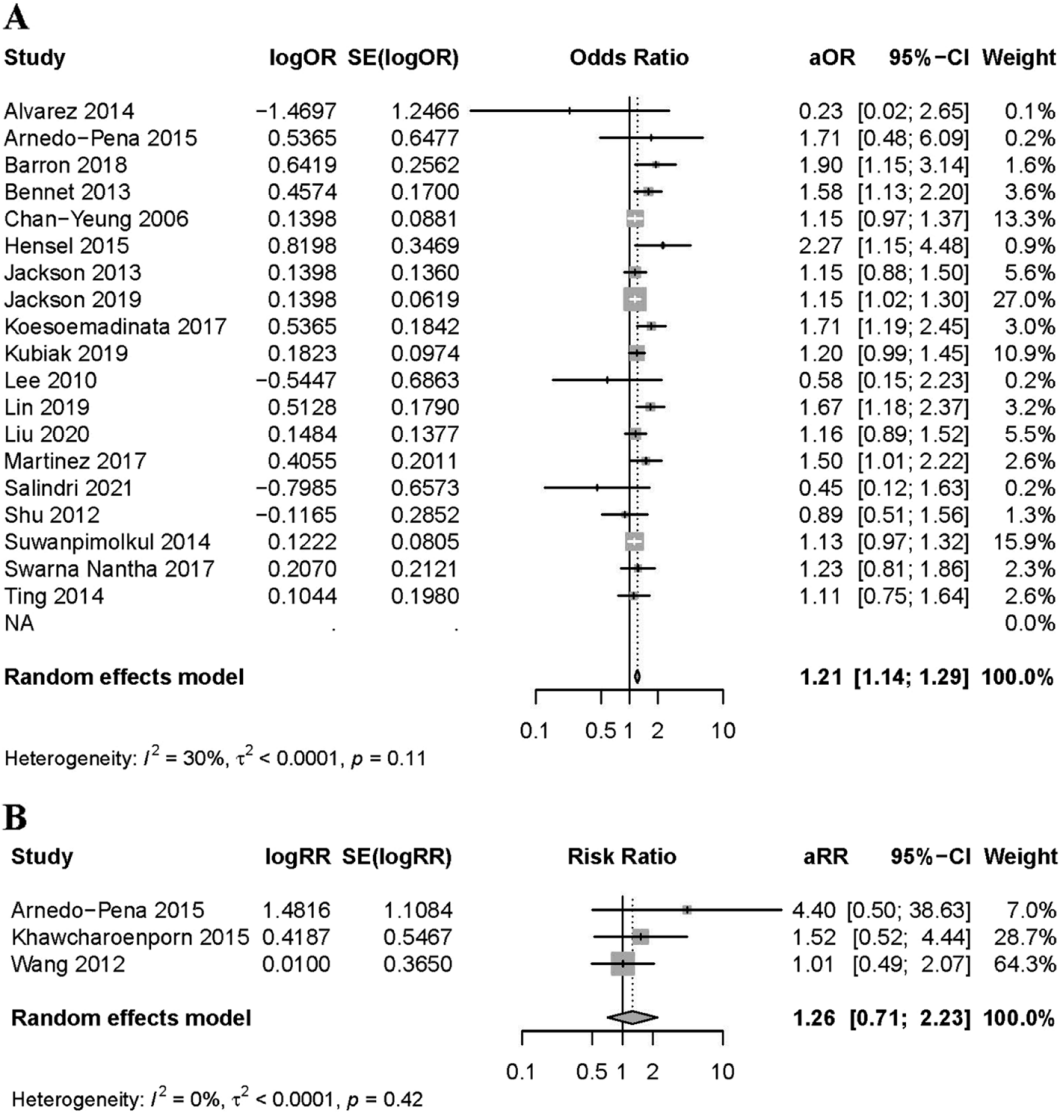
Forest plot of observational studies on diabetes and latent tuberculosis infection: adjusted estimates. Footnote: **A** the forest plot of cross-sectional studies; **B** the forest plot of cohort studies

**Fig. 3:**
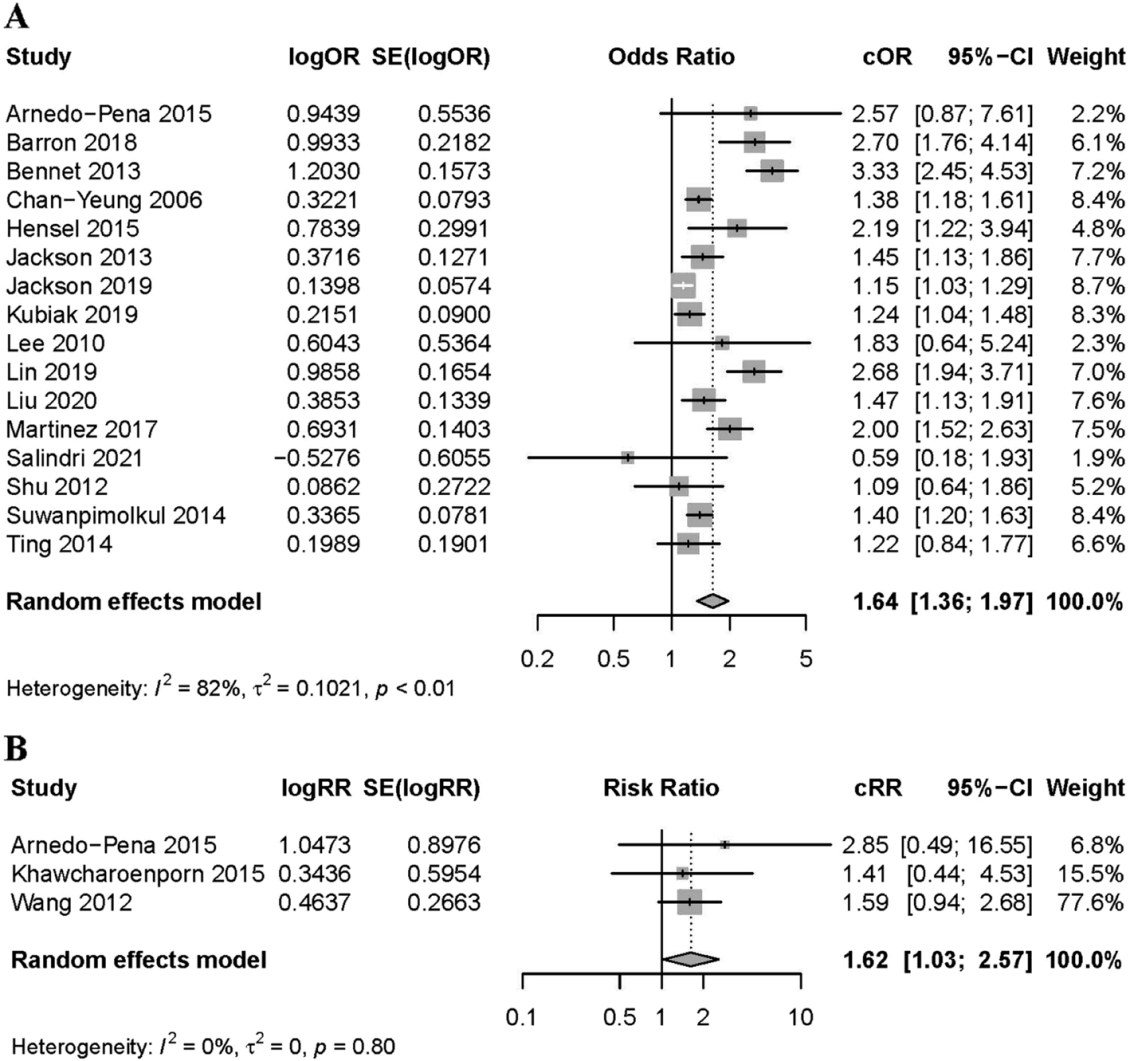
Forest plot of observational studies on diabetes and latent tuberculosis infection: crude estimates. Footnote: **A** the forest plot of cross-sectional studies; **B** the forest plot of cohort studies

The cohort study results showed that the incidence of DM is positively associated to that of LTBI (pooled aRR: 1.26, 95% CI: 0.71–2.23). The pooled aOR of cross-sectional studies was 1.21 (95% CI: 1.14–1.29). Overall, the results of cohort and cross-sectional studies were positively associated with a forest plot (Fig. 2), indicating that DM was a risk factor for LTBI. The pooled estimate of the three crude RR for cohort studies was 1.62 (95% CI: 1.03–2.57). Among the cross-sectional studies included, sixteen studies provided crude OR values, resulting in a pooled cOR of 1.64 (95% CI: 1.36–1.97), also supporting the observed positive correlation. (Fig. 3).

### Subgroup analysis of cross-sectional studies

In order to explore the heterogeneity for the combined adjusted values, we conducted meta-regression and subgroup analysis on a total of 19 cross-sectional studies. We performed subgroup analyses of 19 cross-sectional studies stratified by study population, study region, DM diagnostics, LTBI diagnostics, LTBI prevalence, TB burden, and bias of quality assessment (Table 2). The results of the meta-regression analysis (Supplementary Table S2) demonstrate that within the framework of multivariate analysis, both self-reported diabetes and alternative diagnostic methods (non-self-report) exert notable influences on the outcomes (*pval* < 0.05). The pooled aOR was higher in the DM group using HbA1c diagnosis than in the self-reported DM group (pooled aOR: 1.56, 95% CI: 1.24–1.96 *vs*. 1.17, 95% CI: 1.06–1.28, respectively).

**Table 2:** The subgroup analysis for cross-sectional studies

	Studies (n)	Participants (n)	aOR (95% CI)	*I*^*2*^ (%)	pval*
Population					
Contacts	5	7065	1.26 (1.07–1.50)	28	0.866
Immigrants or refugees	3	5275	1.44 (1.01–2.06)	69	0.861
Immunosuppressed patients	2	4858	0.84 (0.50–1.40)	0	0.127
Others	3	5386	1.15 (1.00–1.34)	0	0.400
Community residents	6	27,294	1.31 (0.09–1.58)	52	Ref
Study region					
Europe	3	14,273	1.15 (1.03–1.29)	0	0.5702
North America	7	36,633	1.41 (1.11–1.77)	57	0.4662
Asia	9	16,446	1.22 (1.11–1.34)	17	Ref
DM diagnostics					
Self-report	6	19,705	1.17 (1.06–1.28)	0	0.0238
Others	8	37,664	1.21 (1.09–1.35)	25	0.0456
HbA1c	5	9983	1.56 (1.24–1.96)	41	Ref
LTBI diagnostics					
TST	4	5666	1.17 (1.04–1.32)	0	0.6171
TST and/or IGRA	4	30,201	1.39 (1.08–1.80)	52	0.5118
IGRA	11	31,485	1.24 (1.10–1.40)	40	Ref
LTBI prevalence					
≥ 30%	6	33,135	1.17 (1.06–1.28)	0	0.2687
< 30%	13	34,217	1.31 (1.15–1.49)	38	Ref
TB burden^a^					
0–30 per 100,000	9	50,520	1.26 (1.11–1.45)	49	0.5536
30–100 per 100,000	8	15,068	1.19 (1.06–1.34)	0	0.4116
> 100 per 100,000	2	1764	1.38 (0.98–1.95)	65	Ref
Risk of bias					
Moderate	3	8227	1.17 (1.01–1.37)	19	0.6465
Low	16	59,125	1.24 (1.14–1.35)	34	Ref

*Abbreviations*: *CI* confidence interval, *IGRA* interferon-γ release assay, *LTBI* latent tuberculosis infection, *DM* diabetes mellitus, *TST* tuberculin skin test

^*^The *p*-values were obtained through a univariate meta-regression

^a^Dates were from WHO website (https://www.who.int/teams/global-tuberculosis-programme/tb-reports)

Regarding the diagnostic methods for LTBI, the aOR results for the TST group and the IGRA group were similar (pooled aOR: 1.17, 95% CI: 1.04–1.32 *vs*. 1.24, 95% CI: 1.10–1.40). Although not reaching statistical significance, the pooled aOR of immunosuppressed patients was lower than that of community residents (pooled aOR: 0.84, 95% CI: 0.50–1.40 *vs*. 1.31, 95% CI: 0.09–1.58). Similarly, the pooled aOR of individuals from areas with a TB burden of more than 100 per 100,000 was higher than that of those from areas with a TB burden less than 100 per 100,000 (Table 2).

### Sensitivity analysis and publication bias

In the sensitivity analyses, the pooled results were consistent. When a single study was sequentially excluded from the analysis, the results showed that DM had an effect on LTBI, suggesting that pooled estimates were stable (Supplementary Figure S3). A funnel plot of ORs of cross-sectional studies showed no evidence of asymmetry via visual inspection (Supplemental Figure S4). The Cochran's Q test (*pval* = 0.18) revealed no statistical evidence of heterogeneity and the Egger’s test (*pval* = 0.515) revealed no statistical evidence of publication bias.

## Discussion

The review included 22 observational studies reporting the association between DM and LTBI with adjusted estimates of effects. The pooled aRR of cohort studies was 1.26 (95% CI: 0.71–2.23) and the pooled aOR of cross-sectional studies was 1.21 (95% CI: 1.14–1.29). Both cohort and cross-sectional studies suggested that DM increases the risk of LTBI and DM is a risk factor for LTBI. The pooled estimate of the crude RR for three cohort studies was 1.62 (95% CI: 1.03–2.57). Among the cross-sectional studies included, sixteen studies provided crude OR values, resulting in a pooled cOR of 1.64 (95% CI: 1.36–1.97), also supporting the observed positive correlation.

A meta-analysis conducted by Lee and colleagues in 2017 and Liu and colleagues in 2022, both revealed a positive association between DM and LTBI [9, 10], consistent with findings in our study. Our study considered the effect of confounding factors on DM and LTBI outcomes, mainly using adjusted effect estimates. Among the 16 included studies providing both rough and adjusted OR values, 14 reported lower adjusted than crude OR values (Supplementary material Table S3), suggesting that the use of unadjusted ORs may lead researchers to overestimate the association between DM and LTBI. Consequently, it is possible that the meta-analysis reported by Liu et al. observed a significant association between DM and LTBI [9] because it was amplified due to the influence of confounding factors influencing rough effect estimates of the association between DM and LTBI. A strength of our study versus that of Liu et al. is that our study used adjusted effect estimates to improve the accuracy of results.

Although not reaching statistical significance (overlapping 95% CIs), our assessment of cohort studies showed a tighter association between DM and LTBI (pooled aRR: 1.26,95% CI: 0.71–2.23) than that of cross-sectional studies (pooled aOR: 1.21, 95% CI: 1.14–1.29). This finding may be due to the missed diagnosis of patients in incubation and remission stages in the cross-sectional studies. A cross-sectional study is a snapshot in which differing results may be observed if a different time frame is chosen [38]; therefore, the missed diagnosis of patients in incubation and remission periods may have affected results. Because LTBI is unlikely to lead to the development of DM, the underdiagnosis of LTBI in diabetic and non-diabetic patients may lead to an underestimation of the association between DM and LTBI. Cohort studies may be used to evaluate the development of an outcome of interest over time, among groups of initially unexposed participants who are and are not exposed throughout the study course [39]. Therefore, cohort studies likely more accurately reflect the causal association between DM and LTBI. The publication of additional cohort studies may provide new evidence regarding the association between DM and LTBI.

We use meta-regression to explore heterogeneity, and the results indicate that within the context of multivariate analysis, both self-reported diabetes and alternative diagnostic methods (non-self-report) significantly impact the outcomes (*pval* < 0.05). Subgroup analyses revealed that the pooled aOR of the HbA1c group was higher than that of self-reported group (pooled aOR: 1.56, 95% CI: 1.24–1.96 *vs*. 1.17, 95% CI: 1.06–1.28). This further confirms the findings of Lee et al. [8]. that screening people with DM for LTBI may have a greater impact on people with DM identified by rigorous diagnostic modalities.

In addition to the diagnostic methods for DM that may affect the results of aRR or aOR, the diagnostic methods for LTBI can also have an impact on these outcomes. However, our subgroup analysis results indicate that whether diagnosing LTBI using TST or IGRA, there is no significant influence on the aOR results. Additionally, the pooled aOR of immunosuppressed patients was lower than that of community residents (pooled aOR: 0.84, 95% CI: 0.50–1.40 *vs*. 1.31, 95% CI: 0.09–1.58), although not reaching statistical significance. Similarly, the pooled aOR of individuals from areas with a TB burden of more than 100 per 100,000 was higher than that of those from areas with a TB burden less than 100 per 100,000. Our results suggest that the relationship between DM and LTBI may be influenced by the patients' immune status and the TB burden of the areas. These results may be attributed to the significant influence of patients' immune status and the TB burden of the areas on the positive rates of IGRA and TST [40]. It's worth noting that our study included only two studies each for the immunosuppressed patients and individuals from areas with a TB burden of more than 100 per 100,000. Therefore, further research is needed to confirm our findings.

This study has several potential limitations. First, only 5 of the 22 included studies used a uniform glycosylated hemoglobin assay to diagnose DM, while the remaining studies relied on self-reported disease or prior hospital records to determine the prevalence of DM. Second, our study primarily aimed to investigate the association between DM and LTBI and did not specifically address cost analysis or the impact of LTBI screening on TB prevention due to the limitations of the study design. Future research should consider conducting more detailed investigations in these areas. Finally, the lack of precision in the adjusted effect estimates can be attributed to the limited number of included cohort studies. Therefore, future research should aim to include a greater number of cohort studies to enhance the robustness of our findings.

## Conclusion

Our systematic review and meta-analysis suggest a positive association between DM and LTBI. Individuals with DM may have a higher risk of LTBI compared to those without DM. These findings provide important insights for future research and public health interventions in managing LTBI in diabetic populations.

### Supplementary Information

**Additional file 1: Figure S1.** The quality evaluation of cross-sectional studies. **Figure S2.** The quality evaluation of cohort studies. **Figure S3.** The sensitivity analysis of the odds ratio for diabetes mellitus and latent tuberculosis infection. **Figure S4.** Funnel plot of observational studies on diabetes mellitus and latent tuberculosis infection. **Table S1.** Search strategies and search results for each database. **Table S2.** Meta-regression of heterogeneity sources in the relationship between DM and LTBI. **Table S3.** Crude and adjusted ORs from 16 cross-sectional studies that reported both crude and adjusted ORs. **Table S4.** The subgroup analysis for 16 cross-sectional studies that reported both crude and adjusted ORs.

**Additional file 2: **Risk of bias of studies involved using the Newcastle-Ottawa quality assessment scale.

## Data Availability

Data may be made available by contacting the corresponding author.

## Abbreviations

LTBI: Latent tuberculosis infection
DM: Diabetes mellitus
OR: Odds ratio
RR: Relative risk
CI: Confidence interval
