# Supplementary material for: Diabetes mellitus and latent tuberculosis infection: an updated meta-analysis and systematic review
Source: BMC Infect Dis. 2023 Nov 8;23:770. doi: 10.1186/s12879-023-08775-y (PMC10631079; doi:10.1186/s12879-023-08775-y)
Supplement: Supplementary file 1 — Additional file 1: Figure S1. The quality evaluation of cross-sectional studies. Figure S2. The quality evaluation of cohort studies. Figure S3. The sensitivity analysis of the odds ratio for diabetes mellitus and latent tuberculosis infection. Figure S4. Funnel plot of observational studies on diabetes mellitus and latent tuberculosis infection. Table S1. Search strategies and search results for each database. Table S2. Meta-regression of heterogeneity sources in the relationship between DM and LTBI. Table S3. Crude and adjusted ORs from 16 cross-sectional studies that reported both crude and adjusted ORs. Table S4. The subgroup analysis for 16 cross-sectional studies that reported both crude and adjusted ORs. [file 12879_2023_8775_MOESM1_ESM.pdf]

Supplementary web appendix for:
Diabetes Mellitus and Latent Tuberculosis Infection: an update meta-analysis and Systematic review

Figure S1. The quality evaluation of cross-sectional studies 2
Figure S2. The quality evaluation of cohort studies 3
Figure S3. The sensitivity analysis of the odds ratio for diabetes mellitus and latent tuberculosis infection. 4
Figure S4. Funnel plot of observational studies on diabetes mellitus and latent tuberculosis infection .. 5
Table S1. Search strategies and search results for each database 6
Table S2. Meta-regression of heterogeneity sources in the relationship between DM and LTBI. 7
Table S3. Crude and adjusted ORs from 16 cross-sectional studies that reported both crude and adjusted ORs. 8
Table S4. The subgroup analysis for 16 cross-sectional studies that reported both crude and adjusted ORs 9

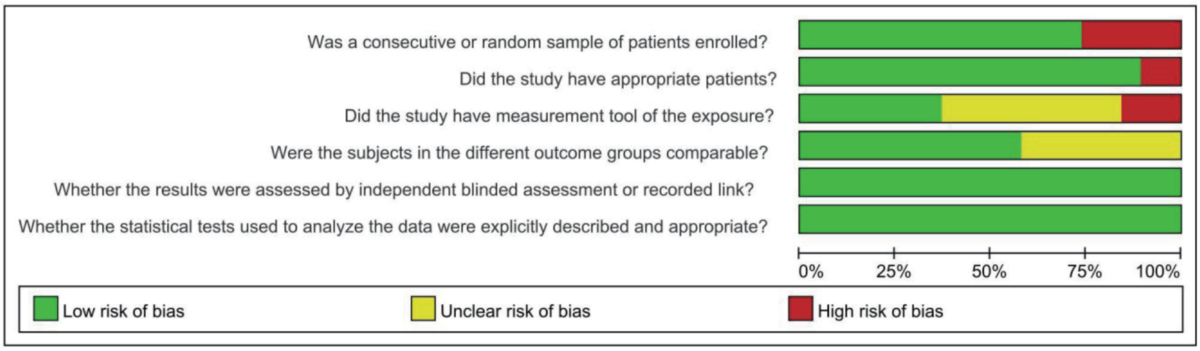

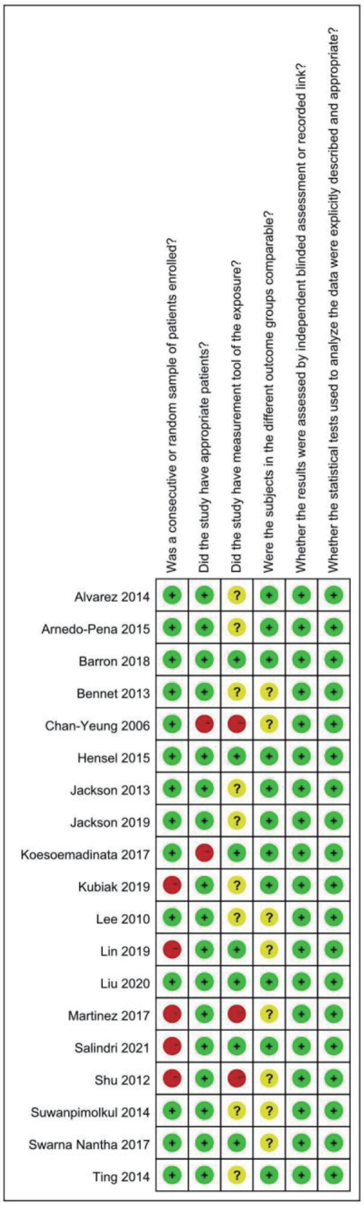

Figure S1. The quality evaluation of cross-sectional studies

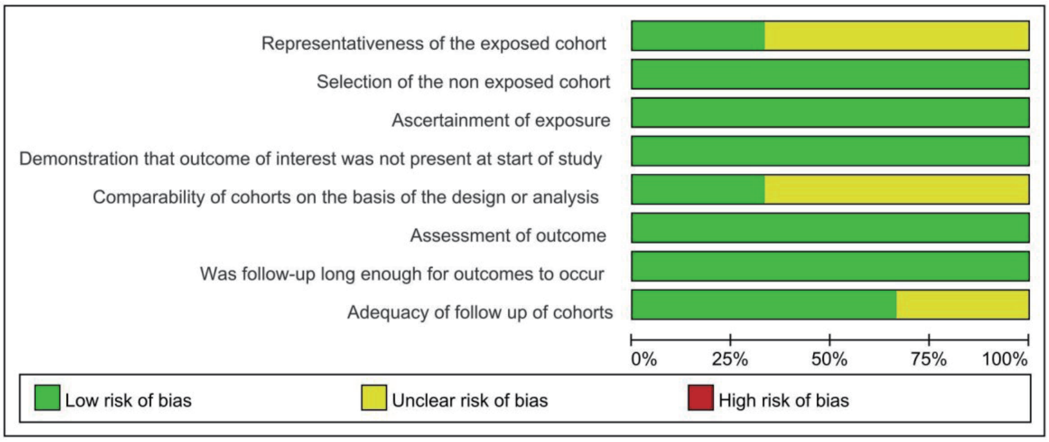

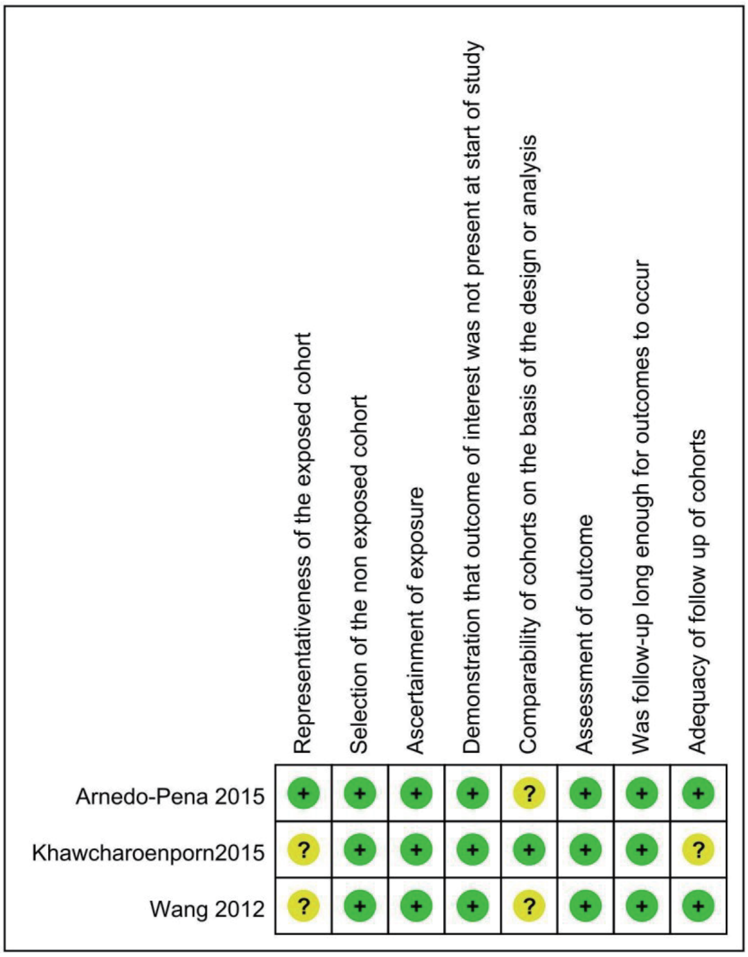

Figure S2. The quality evaluation of cohort studies

Study	aOR	95%−CI	P−value	Tau2	Tau	I2
Omitting Arnedo−Pena 2015	1.24	[1.08; 1.41]	< 0.01	0.0805	0.2838	85%
Omitting Khawcharoenporn 2015	1.25	[1.07; 1.45]	< 0.01	0.0978	0.3127	85%
Omitting Wang 2012	1.27	[1.09; 1.47]	< 0.01	0.0963	0.3103	85%
Omitting Alvarez 2014	1.29	[1.13; 1.47]	< 0.01	0.0743	0.2726	84%
Omitting Arnedo−Pena 2015	1.24	[1.07; 1.44]	< 0.01	0.0948	0.3079	85%
Omitting Barron 2018	1.23	[1.07; 1.41]	< 0.01	0.0821	0.2865	84%
Omitting Bennet 2013	1.24	[1.07; 1.44]	< 0.01	0.0974	0.3121	84%
Omitting Chan−Yeung 2006	1.26	[1.08; 1.47]	< 0.01	0.1028	0.3207	85%
Omitting Hensel 2015	1.23	[1.08; 1.40]	< 0.01	0.0683	0.2614	83%
Omitting Jackson 2013	1.26	[1.08; 1.47]	< 0.01	0.1026	0.3203	85%
Omitting Jackson 2019	1.26	[1.08; 1.47]	< 0.01	0.1029	0.3208	85%
Omitting Koesoemadinata 2017	1.23	[1.07; 1.43]	< 0.01	0.0916	0.3027	83%
Omitting Kubiak 2019	1.26	[1.08; 1.47]	< 0.01	0.1038	0.3222	85%
Omitting Lee 2010	1.29	[1.13; 1.47]	< 0.01	0.0753	0.2744	85%
Omitting Lin 2019	1.24	[1.07; 1.43]	< 0.01	0.0935	0.3058	83%
Omitting Liu 2020	1.26	[1.08; 1.47]	< 0.01	0.1029	0.3207	85%
Omitting Martinez 2017	1.24	[1.07; 1.45]	< 0.01	0.1001	0.3164	85%
Omitting Salindri 2021	1.30	[1.15; 1.47]	< 0.01	0.0605	0.2460	84%
Omitting Shu 2012	1.28	[1.11; 1.48]	< 0.01	0.0870	0.2950	85%
Omitting Suwanpimolkul 2014	1.26	[1.08; 1.47]	< 0.01	0.1023	0.3198	85%
Omitting Swarna Nantha 2017	1.26	[1.08; 1.46]	< 0.01	0.1033	0.3215	85%
Omitting Ting 2014	1.26	[1.08; 1.47]	< 0.01	0.1011	0.3179	85%
**Random effects model**	**1.26**	**[1.09; 1.45]**	**< 0.01**	**0.0907**	**0.3012**	**85%**

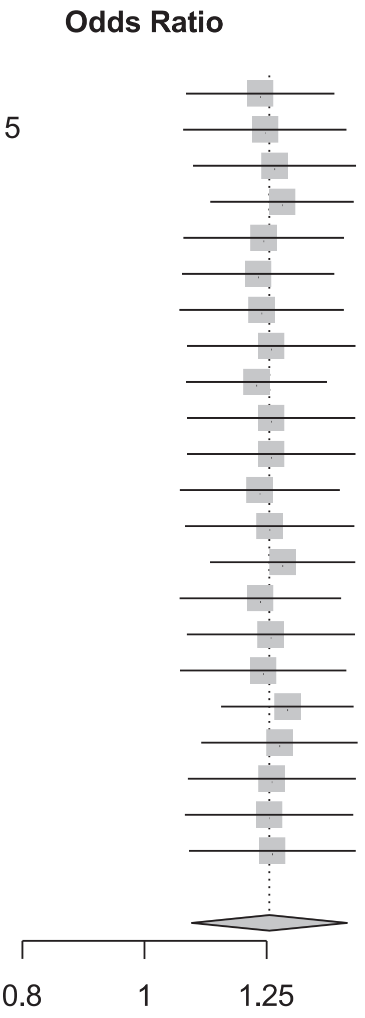

Figure S3. The sensitivity analysis of the odds ratio for diabetes mellitus and latent tuberculosis infection.

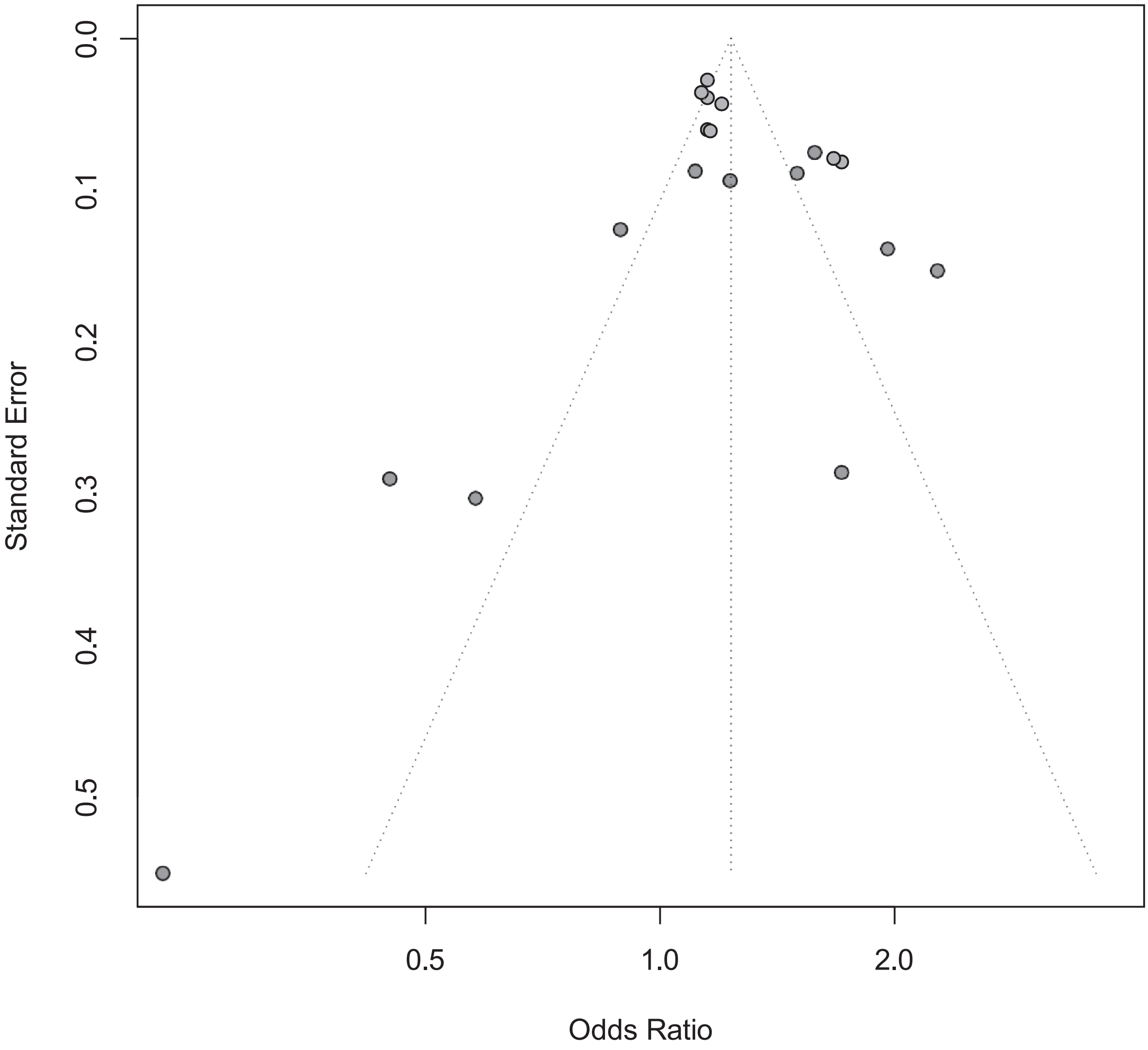

Figure S4. Funnel plot of observational studies on diabetes mellitus and latent tuberculosis infection

Table S1. Search strategies and search results for each database

Database	Search Strategies	Search results
PubMed	(((((Latent Tuberculosis Infections[MeSH Terms]) OR (Latent Tuberculosis)) OR (LTBI)) OR (Tuberculosis Infections)) OR (Tuberculosis)) AND (((Diabetes Mellitus[MeSH Terms]) OR (Diabetes)) OR (DM))	5439 studies
Embase	#1 'latent tuberculosis infections' OR 'latent tuberculosis':ti,ab,kw OR ltbi:ti,ab,kw OR 'tuberculosis infections':ti,ab,kw OR tuberculosis:ti,ab,kw<br>#2 'diabetes mellitus'/exp OR diabetes:ti,ab,kw OR dm:ti,ab,kw<br>#3 #1 AND #2	7332 studies
Cochrane Library	#1 MeSH descriptor: [Latent Tuberculosis] this term only<br>#2 (Latent Tuberculosis Infections):ti,ab,kw OR (LTBI):ti,ab,kw OR (Tuberculosis Infections):ti,ab,kw OR (Tuberculosis):ti,ab,kw<br>#3 MeSH descriptor: [Diabetes Mellitus] this term only #4 (Diabetes):ti,ab,kw OR (DM):ti,ab,kw<br>#5 (#1 OR #2) AND (#3 OR #4)	288 studies

Table S2. Meta-regression of heterogeneity sources in the relationship between DM and LTBI.

	Univariate analysis			Multivariate analysis	
	pval	Estimat (95%CI)	R^2 (%)	*pval*	Estimat (95%CI)
TB burden			0.00		
0–30	0.5536	-0.0710 (-0.3059 to 0.1639)		0.2394	-0.9668 (-1.5773 to 0.6437)
30–100	0.4116	-0.1040 (-0.3522 to 0.1442)		0.4964	-0.2447 (-0.9500 to 0.4605)
> 100	Ref	Ref		Ref	Ref
Population			0.00		
Contacts	0.8659	-0.0231 (-0.2909 to 0.2447)		0.8879	-0.0272 (-0.4058 to 0.3514)
Immigrants or Refugees	0.8608	0.0256 (-0.2600 to 0.3111)		0.1233	0.6981 (-0.1899 to 1.5806)
Immunosuppressed	0.1269	-0.4552 (-1.0396 to 0.1293)		0.0387	-0.9520 (-1.8545 to -0.0494)
Community residents	Ref	Ref		Ref	Ref
Others	0.3997	-0.1214 (-0.4038 to 0.1611)		0.4532	-0.2508 (-0.9064 to 0.4047)
Region			0.00		
Europe	0.5702	-0.0608 (-0.2707 to 0.1491)		0.5158	0.4671 (-0.9418 to 1.8760)
North America	0.4662	0.0760 (-0.1284 to 0.2804)		0.6924	0.2909 (-1.1505 to 1.7323)
Asia	Ref	Ref		Ref	Ref
DM diagnostics			77.33		
Self-report	0.0238	-0.2828 (-0.5279 to -0.0376)		0.1689	-0.3691 (-0.8948 to 0.1567)
Others	0.0456	-0.2514 (-0.4978 to -0.0050)		0.0621	-0.4203 (-0.8619 to 0.0213)
HbA1c	Ref	Ref		Ref	Ref
LTBI diagnostics			0.00		
TST	0.6171	-0.0530 (-0.2610 to 0.1549)		0.4213	-0.2457 (-0.8447 to 0.3532)
TST or IGRA	0.5118	0.0725 (-0.1442 to 0.2892)		0.3060	-0.2552 (-0.7439 to 0.2334)
IGRA	Ref	Ref		Ref	Ref
LTBI prevalence			0.00		
< 30%	Ref	Ref		Ref	Ref
≥ 30%	0.2687	-0.0816 (-0.2262 to 0.0630)		0.2570	-0.2062 (-0.5764 to 0.1640)
Bias			0.00		
Low	Ref	Ref		Ref	Ref
Moderate	0.6465	-0.0428 (-0.2257 to 0.1401)		0.0361	0.4715 (0.0305 to 0.9125)

Abbreviations: TB, tuberculosis; IGRA, interferon-γ release assay; LTBI, latent tuberculosis infection; DM, diabetes

Table S3. Crude and adjusted ORs from 16 cross-sectional studies that reported both crude and adjusted ORs.

Author and year	cOR (95% CI)	aOR (95% CI)	Adjusted Variables
Arnedo-Pena 2015[17]	2.57 (0.87–7.62)	1.71 (0.48–6.08)	Age, sex, smoking
Barron 2018[21]	3.12 (1.62–6.02)	1.96 (1.06–3.63)	Age, sex, smoking status, history of active TB, and foreign-born status
Bennet 2013[22]	3.33 (2.44–4.52)	1.58 (1.13–2.20)	Birth region, age, gender, education, malignancy, HIV, end–stage renal disease, smoking
Chan-Yeung 2006[23]	1.38 (1.18–1.61)	1.15 (0.97–1.37)	Age, marital status, education, place of birth, smoking, drug abuse, past tuberculosis, cardiovascular disease, arthritis, fracture, ischemic heart disease, COPD, cancer, liver disease, BMI, feeding method, Norton score
Hensel 2015[24]	2.19 (1.22–3.94)	2.27 (1.15–4.48)	Age, sex, BMI, TB incidence in country of origin, smoking status, and vitamin D level
Jackson 2013[25]	1.45 (1.13–1.86)	1.15 (0.88–1.50)	Age (further adjustment for sex, ethnicity, birthplace and the TB exposure history which did not change the estimate)
Jackson 2019[26]	1.15 (1.03–1.29)	1.15 (1.02–1.30)	Sex, age group, ethnicity, immunosuppression and BMI
Kubiak 2019[28]	1.24 (1.04–1.48)	1.20 (0.99–1.45)	Age, sex, body mass index category, smoking, and hazardous alcohol use
Lee 2010[29]	1.83 (0.64–5.24)	0.58 (0.15–2.21)	Age, gender, dialysis vintage
Lin 2019[30]	2.68 (1.94–3.71)	1.67 (1.18–2.38)	Age, gender, smoking status, chronic kidney disease, history of TB and TB contact in this multivariable logistic regression model
Liu 2020[31]	1.47 (1.13–1.91)	1.16 (0.88–1.51)	Age, sex, body mass index, smoking status, number of BCG scars
Martinez 2017[32]	2.00 (1.50–2.60)	1.50 (1.00–2.20)	Participant age, gender, TB exposure history of household, birthplace, smoking status, family size, and diabetes status
Salindri 2021[33]	0.59 (0.19–2.04)	0.45 (0.13–1.71)	Age and sex
Shu 2012[34]	1.09 (0.64–1.86)	0.89 (0.51–1.56)	Age, gender, old tuberculosis, smoking
Suwanpimolkul 2014[35]	1.40 (1.20–1.63)	1.13 (0.97–1.33)	Age (cut–point, 45 years), HIV status, place of birth
Ting 2014[37]	1.22 (0.84–1.77)	1.11 (0.75–1.63)	Age, sex, BCG vaccination, smoking, COPD, fibrocalcified lesion in chest plain film

Abbreviations: BCG, bacillus calmette–guerin; BMI, body mass index; CI, confidence interval; COPD: chronic obstructive pulmonary disease; HIV, human immunodeficiency virus.

Table S4. The subgroup analysis for 16 cross-sectional studies that reported both crude and adjusted ORs

	Studies (n)	Participants (n)	DM LTBI rate(%)	I^2 (%)	Non-DM LTBI rate(%)	I^2 (%)	Crude OR	I^2 (%)	Adjusted OR	I^2 (%)
**All study**	16	57,247		99	0.28 (0.25–0.31)	100	1.64 (1.36–1.97)	82	1.20 (1.12–1.28)	26
**Population**										
Contacts	3	13,903		96	0.41 (0.18–0.65)	100	1.33 (1.14–1.55)	1	1.19 (1.02–1.39)	0
Community residents	6	35,421		96	0.15 (0.06–024.)	90	1.72 (1.20–2.47)	92	1.31 (1.09–1.58)	52
Immigrants or Refugees	3	1589		43	0.29 (0.00–0.65)	87	2.14 (1.25–3.67)	0	1.44 (1.01–2.06)	69
Immunosuppressed	2	4589		0	0.15 (0.01–0.30)	98	1.21 (0.75–1.59)	0	0.84 (0.50–1.14)	0
Others	2	1170		100	0.33 (0.01–0.66)	44	1.36 (1.17–1.56)	51	1.14 (0.98–1.34)	0
**TB burden**										
0–30	8	37,173		100	0.26 (0.10–0.43)	100	1.76 (1.30–2.37)	89	1.18 (1.05–1.34)	5
30–100	7	18,631		89	0.21 (0.05–0.37)	100	1.57 (1.20–2.04)	65	1.28 (1.11–1.47)	49
>100	1	4958		Ref	0.54 (0.51–0.97)	Ref	1.24 (1.04–1.48)	Ref	1.20 (0.99–1.45)	Ref
**Region**										
Asia	7	18,252		94	0.28 (0.11–0.45)	100	1.47 (1.17–1.84)	68	1.19 (1.07–1.32)	1
Europe	3	10,749		0	0.19 (0.01–0.37)	100	1.30 (1.03–1.65)	57	1.15 (1.03–1.29)	0
North America	6	27,971		100	0.26 (0.03–0.48)	100	2.02 (1.43–2.85)	85	1.43 (1.13–1.81)	59
**DM diagnostics**										
HbA1c	4	26,646		92	0.15 (0.06–0.23)	95	2.46 (1.95–3.10)	51	1.71 (1.32–2.22)	40
Self-report	6	14,718		98	0.23 (0.08–0.38)	100	1.37 (1.12–1.67)	68	1.17 (1.06–1.28)	0
Others	6	15,608		99	0.35 (0.11–0.60)	100	1.67 (1.21–2.30)	83	1.18 (1.07–1.30)	0
**LTBI diagnostics**										
IGRA	10	43,458		97	0.18 (0.12–0.25)	100	1.51 (1.23–1.85)	72	1.18 (1.08–1.30)	31
TST or IGRA	4	12,546		100	0.27 (0.00–0.63)	100	2.31 (1.47–3.62)	91	1.39 (1.08–1.80)	52
TST	2	968		98	0.59 (0.49–0.69)	97	1.32 (1.17–1.48)	0	1.17 (1.03–1.33)	0
**LTBI prevalence**										
< 30%	10	29,602		97	0.16 (0.10–0.23)	100	1.74 (1.30–2.33)	88	1.29 (1.12–1.48)	38
≥ 30%	6	27,370		100	0.41 (0.17–0.65)	100	1.38 (1.26–1.50)	0	1.17 (0.06–1.28)	0
**Bias**										
Low	13	51,184		99	0.25 (0.13–0.37)	100	1.68 (1.34–2.11)	84	1.20 (1.12–1.29)	32
Moderate	3	5788		99	0.30 (0.00–0.65)	100	1.50 (1.10–2.05)	70	1.17 (1.01–1.37)	19

Abbreviations: CI, confidence interval; IGRA: interferon-γ release assay; LTBI, latent tuberculosis infection; DM, diabetes mellitus; TST, tuberculin skin test.
