# Supplementary material for: Diabetes mellitus and latent tuberculosis infection: an updated meta-analysis and systematic review
Source: BMC Infect Dis. 2023 Nov 8;23:770. doi: 10.1186/s12879-023-08775-y (PMC10631079; doi:10.1186/s12879-023-08775-y)
Supplement: Supplementary file 2 — Additional file 2: Risk of bias of studies involved using the Newcastle-Ottawa quality assessment scale. [file 12879_2023_8775_MOESM2_ESM.pdf]

Supplementary material 2. Risk of bias of studies involved using the Newcastle-Ottawa quality assessment scale

Cross sectional studies									
	Selection				Comparability	Outcome			
first author	1)	2)	3)		4)	5)	6)	scores	risk of bias
Alvarez 2014	1	1	0		2	1	1	6	Low
Arnedo-Pena 2015	1	1	0		2	1	1	6	Low
Barron 2018	1	1	1		2	1	1	7	Low
Bennet 2013	1	1	0		1	1	1	5	Low
Chan-Yeung 2006	1	0	0		1	1	1	4	Moderate
Hensel 2016	1	1	1		2	1	1	7	Low
Jackson 2013	1	1	0		2	1	1	6	Low
Jackson 2019	1	1	0		2	1	1	6	Low
Koesoemadinata 2017	1	0	1		2	1	1	6	Low
Kubiak 2019	0	1	0		2	1	1	5	Low
Lee 2010	1	1	0		1	1	1	5	Low
Lin 2019	0	1	1		1	1	1	5	Low
Liu 2020	1	1	1		2	1	1	7	Low
Martinez 2017	0	1	0		1	1	1	4	Moderate
Salindri 2021	0	1	1		2	1	1	6	Low
Shu 2012	0	1	0		1	1	1	4	Moderate
Suwanpimolkul 2014	1	1	0		1	1	1	5	Low
Swarna Nantha 2017	1	1	1		1	1	1	6	Low
Ting 2014	1	1	0		2	1	1	6	Low

Cohort studies										
	Selection				Comparability	Outcome				
first author	1)	2)	3)	4)	5)	6)	7)	8)	scores	risk of bias
Arnedo-Pena 2015	1	1	1	1	1	1	1	1	8	Low
Wang 2012	0	1	1	1	2	1	1	0	7	Low
Khawcharoenporn 2015	0	1	1	1	1	1	1	1	7	Low

For cross sectional studies:

Selection

1) Was a consecutive or random sample of patients enrolled? (We scored “1” if a consecutive or random sample of eligible patients was enrolled; “0” if patients were selected by convenience;)

2) Did the study have appropriate patients? (We scored “1” if current active TB and people with TB symptom were excluded, or they were grouped separately, or the definition of LTBI was stated as asymptomatic; “0” if active TB and people with TB symptom were not excluded;)

3) Did the study have measurement tool of the exposure(We scored “1” if the study had measurement tool of diabetes mellitus; “0” if the study hadn’t measurement tool of diabetes mellitus;)

Comparability
4) The subjects in different outcome groups are comparable, based on the study design or analysis. Confounding factors are controlled.(We scored “2” if the confounding adjusted for sex ; “1” if the confounding adjusted for others;)

Outcome
5) Assessment of the outcome(We scored “1” if the outcome was assesseed by independent blind assessment or record linkage; “0” if the outcome was assesseed by self report or no description)
6) Statistical test(We scored “1” if the statistical test used to analyze the data is clearly described and appropriate, and the measurement of the association is presented, including confidence intervals and the probability level;“0” if the statistical test is not appropriate, not described or incomplete)

For cohort studies(We record the single evaluation as 1 point if the study satisfies the description with an asterisk in the individual evaluation):
1) Representativeness of the exposed cohort
*a) truly representative of the average _______________ (describe) in the community
*b) somewhat representative of the average ______________ in the community
c) selected group of users eg nurses, volunteers
d) no description of the derivation of the cohort
2) Selection of the non exposed cohort
*a) drawn from the same community as the exposed cohort
b) drawn from a different source
c) no description of the derivation of the non exposed cohort
3) Ascertainment of exposure
*a) secure record (eg surgical records)
*b) structured interview
c) written self report
d) no description
4) Demonstration that outcome of interest was not present at start of study
*a) yes
b) no
5) Comparability of cohorts on the basis of the design or analysis
*a) study controls for the most important factor)
*b) study controls for any additional factor (This criteria could be modified to indicate specific control for a second important factor.)
6) Assessment of outcome
*a) independent blind assessment
*b) record linkage
c) self report
d) no description
7) Was follow-up long enough for outcomes to occur
*a) yes (select an adequate follow up period for outcome of interest)

b) no

8) Adequacy of follow up of cohorts

*a) complete follow up - all subjects accounted for

*b) subjects lost to follow up unlikely to introduce bias - small number lost - > 70 % follow up, or description provided of those lost)

c) follow up rate < 70% and no description of those lost

d) no statement
